# Extracellular Microbial Metabolomics: The State of the Art

**DOI:** 10.3390/metabo7030043

**Published:** 2017-08-22

**Authors:** Farhana R. Pinu, Silas G. Villas-Boas

**Affiliations:** 1 The New Zealand Institute for Plant & Food Research Limited, Private Bag 92169, Auckland 1142, New Zealand; 2 School of Biological Sciences, University of Auckland, Private Bag 92019, Auckland 1010, New Zealand; s.villas-boas@auckland.ac.nz

**Keywords:** metabolite footprinting, exometabolome, culture medium, metabolic modelling, sample preparation, analytical instruments, data integration, extracellular metabolites, intracellular metabolites

## Abstract

Microorganisms produce and secrete many primary and secondary metabolites to the surrounding environment during their growth. Therefore, extracellular metabolites provide important information about the changes in microbial metabolism due to different environmental cues. The determination of these metabolites is also comparatively easier than the extraction and analysis of intracellular metabolites as there is no need for cell rupture. Many analytical methods are already available and have been used for the analysis of extracellular metabolites from microorganisms over the last two decades. Here, we review the applications and benefits of extracellular metabolite analysis. We also discuss different sample preparation protocols available in the literature for both types (e.g., metabolites in solution and in gas) of extracellular microbial metabolites. Lastly, we evaluate the authenticity of using extracellular metabolomics data in the metabolic modelling of different industrially important microorganisms.

## 1. Introduction

Extracellular metabolomics is the study of low molecular weight extracellular metabolites that are secreted by microbial cells into their environment, namely the culture media. The entire complement of these metabolites is often referred to as the exometabolome. It includes all of the metabolites secreted outside the microbial cell, as well as those components of the microbial culture media that are found in the supernatant [1].

Analysis of the exometabolome yields information about microbial activities under different culture conditions which, coupled with the intracellular metabolic profile, provides a comprehensive overview of microbial metabolism. There are some advantages of extracellular metabolite analysis (metabolic footprinting) over the analysis of intracellular metabolites (metabolic fingerprinting). The separation of extracellular metabolites from microbial cells and their intracellular metabolites can be achieved by simple techniques such as centrifugation and filtration, while the extraction of intracellular metabolites from microbial cells is a complex process [2,3]. Sample preparation and the handling of extracellular samples are also comparatively easier than intracellular sample preparation. Necessary precautions do, however, need to be taken to avoid cross-contamination between intra- and extracellular metabolites [4].

Extracellular metabolites are mainly produced as by-products of the metabolic activities of microorganisms growing in a specific environment. There are various environmental factors which can affect the uptake and secretion of metabolites from and into their environment (i.e., culture medium) such as temperature, pH, the concentration of nutrients, and others. Consequently, the level of extracellular metabolites can be easily modified by changing any one, or more, of these factors [5]. Exometabolome analysis, or metabolic footprint analysis, is the global identification and quantification of the metabolites present in the spent culture medium of microbial cells using different analytical techniques. As the name suggests, the metabolic footprint is the microorganism’s footprint in the extracellular medium due to the cells' uptake of nutrients and secretion of other metabolites during growth [6]. This approach is both qualitative and semi-quantitative [5,7].

### 1.1. Applications and Implications of Metabolic Footprinting

Metabolic footprint analysis provides valuable information about the metabolism of different microorganisms that change in response to different environmental conditions. Therefore, this approach has been extensively applied in different areas, such as bioprocess monitoring, bioremediation, biomarker discovery, and functional genomics. Due to the rapid development of different technical platforms, the popularity of extracellular metabolite analysis has increased considerably in the last ten years. In the near future, analysis of extracellular metabolites will provide more substantial biological information because of the rapid advancement and accessibility of a wide range of different analytical approaches.

The availability of different analytical tools and high-throughput methods has made extracellular metabolite analysis an invaluable technique for monitoring bioprocesses. These technologies have been shown to be useful for the quality control of fermentation processes since they enable the monitoring of changes in metabolite levels during fermentation [5,8]. Metabolic footprinting has been used, for example, to study heterologous protein production [9,10], identify fermentation biomarkers [11], monitor wine aroma development [12,13], and to study metabolic interactions among different yeast strains [14].

In addition to monitoring bioprocesses, metabolic footprint analysis has also been used to monitor and assess the mineralization process of xenobiotic compounds (e.g., petroleum and pesticides), both in the environment [15] and in the laboratory [16]. It is desirable, in this process, to determine whether these compounds are undergoing complete degradation (to CO_2_ and H_2_O) or producing hazardous and more recalcitrant compounds [3]. Potentially, metabolic footprint analysis can be used to identify microorganisms which are able to mineralize xenobiotic compounds. Furthermore, if those microorganisms are pathogenic to humans and plants, it might be possible to employ metabolic engineering techniques to reconstruct new species or to transfer the required genes to non-pathogenic microorganisms [5,17].

Besides the usefulness of extracellular metabolomics in important areas like bioprocess and bioremediation, the integration of metabolic footprinting with other “omics” (i.e., proteomics and genomics) has also been undertaken successfully. These integrated approaches were applied efficiently for the identification and characterization of unknown genes responsible for the production of important enzymes involved in expedient and novel pathways. For instance, there is currently great interest in the industrial production of ethanol (biofuel) from agricultural waste [18,19,20]. This conversion is achieved by completely degrading plant lignocelluloses into fermentable sugars. Panagiotou et al. [21] studied the simultaneous saccharification and fermentation of cellulose to ethanol in a one-step process by using *Fusarium oxysporum*. They identified extracellular organic and amino acids, and found that the activation of the GABA shunt caused the inhibition of the fungus’ Kreb’s cycle. Using both metabolic footprinting and fingerprinting, they described a novel fungal metabolic pathway (Phosphoketolase pathway) involved in xylose metabolism [22,23]. There are a number of other studies that show the value of metabolic footprinting in unraveling the degradation processes of lignocelluloses and monitoring the fermentation behavior of some industrially important microorganisms [24,25,26].

Metabolic footprinting has also gained popularity in microbial functional genomics because of its utility in discriminating between microbial strains. It has been used, for instance, to discriminate between yeast strains by identifying extracellular metabolites related to insertion and deletion mutations [27,28]. In addition, mutant bacterial strains (*Escherichia coli* tryptophan mutants) have also been differentiated after analyzing the exometabolome [4]. This approach, combined with genomics data, is also a powerful and low-cost tool for characterizing and phenotyping single deletion mutants. For example, the phenotypic characterization of transposon inserted mutants of xylanolytic and proteolytic rumen bacteria *Clostridium proteoclasticum* B316^T^ has been shown by analyzing extracellular metabolites [29].

Furthermore, analysis of extracellular metabolites that are secreted by the microorganisms into the culture media allows the identification of quorum sensing (QS) molecules, (i.e., compounds used in cell-to-cell communication in microbial communities) [30,31]. These metabolites need to be analyzed during the different growth phases of the microbial community to determine when the QS molecules are produced. *Pseudomonas aeruginosa* can cause a chronic lung infection by producing QS molecules that enable this microorganism to form biofilm and develop a resistance to antibiotics [32]. Microbial footprinting is a potential approach to developing drugs which inhibit the production of QS molecules in such pathogenic microorganisms [5].

Given that metabolic footprinting has already been successfully applied to various types of biotechnological research, this approach has already proved to be very powerful in unraveling innovative and novel information [9,29,33,34,35,36]. However, the analysis of extracellular metabolites cannot provide evidence about what is happening inside the cell [37], although recently a few software packages have become available to predict the intracellular metabolic statuses of cells using concentrations of extracellular metabolites [38,39]. However, to generate a holistic picture of cell metabolism, the application of both intracellular and extracellular metabolomics is highly recommended.

### 1.2. Dynamic or Time-Resolved Metabolic Footprinting

Recently, dynamic or time-resolved metabolic footprinting has gained considerable interest from the scientific community because this approach allows a better understanding of metabolites secreted by different microbial cells at different time intervals due to the changes in environmental conditions [40,41]. Traditional metabolite footprinting mostly relies on single time point data, thus the analysis of metabolites depends on the microbial growth rate in addition to other factors (e.g., inoculum size). On the other hand, dynamic metabolite footprinting gathers data on microbial growth at different time points [41]. Therefore, this approach is more data-rich and informative than the single point extracellular metabolite analysis [40], thus being an important tool for in-depth phenotyping.

**Figure 1:**
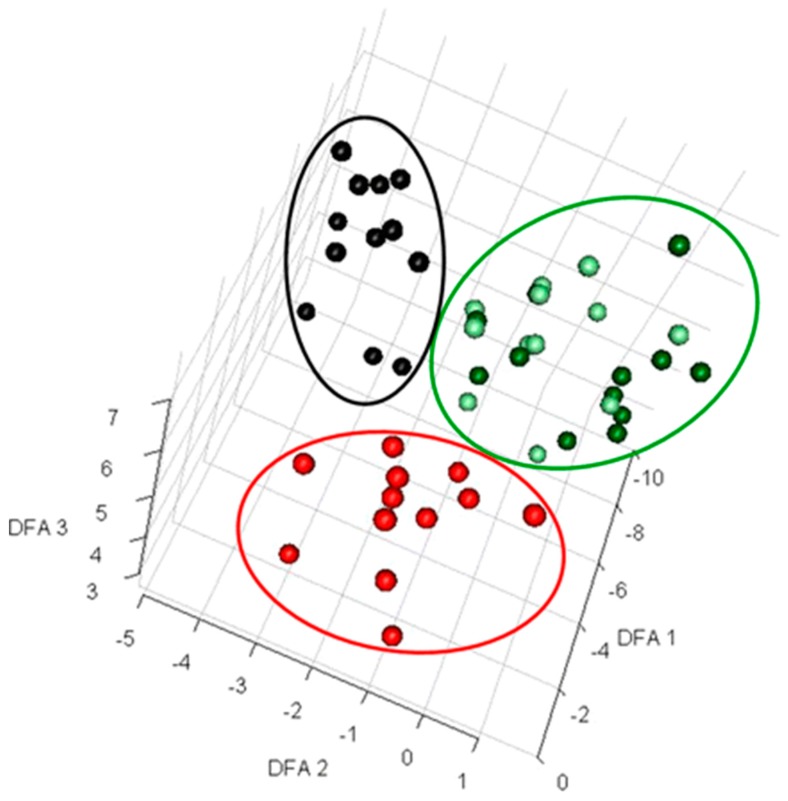
Discriminant function analysis (DFA) to visualize exometabolomics data. Extracellular metabolites from deliberately contaminated and control samples from microalgal fermentation were analyzed using Gas-chromatography and mass spectrometry (GC-MS) [42]. The data from 56 samples were log transformed prior to performing DFA and three distinct clusters were observed. Here, black circles represent the samples from flasks contaminated with *Pseudomonas aeruginosa,* red circles show the samples contaminated by *Bacillus subtilis*, and light green and dark green circles present the samples collected from contaminated flasks at time 0 and non-contaminated flasks, respectively. This figure was reproduced from Sue et al. with the authors’ permission [42].

The changes in growth media due to the presence of contaminants can also be easily determined by analyzing samples at different time intervals rather than at a single point. For example, Sue et al. [42] reported a proof of concept study where they used time series extracellular metabolomics data to detect microbial contamination in microalgal fermentation broths (Figure 1). These broths were deliberately contaminated with common contaminating microorganisms and monitored for nine hour periods at different time intervals to determine the changes in extracellular metabolite profiles. While traditional microbial detection and identification techniques can take two days, this type of metabolite footprinting provides a rapid and conclusive determination of microbial contaminants within a few hours in a batch fermentation process (Figure 1) [42]. Therefore, interventions can be undertaken sooner to avoid the further loss of product quality.

The main benefit of the dynamic or time-resolved metabolic footprinting approach is the ability to capture the changes in the metabolism of microorganisms with different growth rates (especially mutants). Therefore, it is possible to discriminate between mutants and also among the same microbial species. Behrends et al. [43] successfully used this approach to determine the differences between two closely related species of the *Burkholderia cepacia* complex. They also employed a similar approach to determine the differences between *Pseudomonas aeruginosa* wild type and its mutant (mucA22), and identified metabolites associated with osmotic tolerance (e.g., glutamate, trehalose, and glycine-betaine) that caused the main difference between wild-type and mutant strains [43].

The data analysis platform for handling large datasets obtained from dynamic or time-resolved metabolic footprinting has also been updated in recent years [41,44,45]. For instance, Chumnanpuen et al. [41] reported a novel data analysis method (cherry picking and correlation analysis) of MS data based on the identification of significantly correlated metabolites over time, which corresponds to flux ratios of different metabolites. This was followed by a reconstruction of interaction maps to provide more information on metabolic pathways associated with correlated metabolites, thus showing the importance of this approach in generating novel information relating to microbial metabolism [41].

## 2. Sample Preparation for the Analysis of Extracellular Metabolites

**Figure 2:**
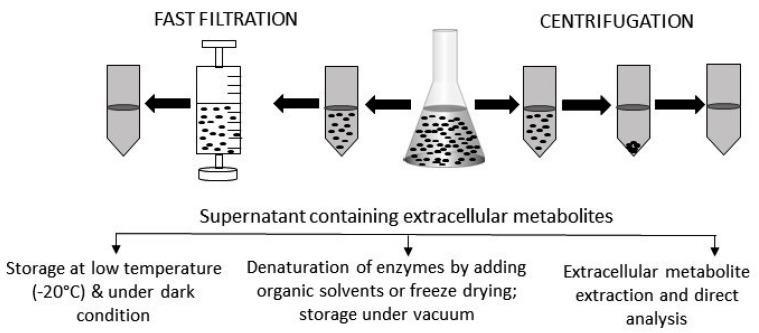
Extracellular sample preparation, handling, and storage. After centrifugation or fast filtration, culture media containing extracellular metabolites are usually stored at a low temperature and under dark conditions. Sometime, organic solvents are added to denature active enzymes. For some metabolites, specific extraction procedures need to be followed before being analyzed by appropriate instruments. Prior to analysis, extracellular samples are often freeze-dried to concentrate the level of metabolites.

Sample preparation is a crucial part of any experimental procedure. In metabolomic analyses the first, and perhaps most critical, step is quenching to halt any biochemical reactions beyond the time of sampling. The microbial culture is then separated from the cells by centrifugation or filtration (Figure 2). The next step is the addition of internal standards that allow the detection and compensation of the technical variability during the sample preparation [46,47]. Moreover, to deactivate the enzymatic activity and concentrate the extracellular metabolites, the culture supernatant must be freeze-dried under vacuum before chemical derivatization. Any remaining culture medium can be stored in the dark at a low temperature (−20 °C or less).

Prior to performing experiments, several factors need to be considered carefully. Especially, the numbers of inoculated cells relative to culture medium volume is particularly of interest so that metabolites are secreted into the surrounding environment in a detectable amount. Microbial cells in a concentration of 10^5^–10^6^ C.F.U per mL are usually recommended for inoculation during extracellular metabolite analysis [42,48]. Moreover, it is also crucial to analyze the unspent culture medium as the control to determine the non-biological changes that occur during the experiments [49].

Extracellular metabolites can be present in solution or in the gas-phase [6]. The sample preparation varies considerably based on the state of the metabolites and the major considerations during the extraction and analysis of metabolites in a liquid or gaseous state will be outlined below:

### 2.1. Metabolites in Solution

Extracellular metabolites are present in the microbial culture media. The composition of liquid media and the possible presence of interferences (matrix), such as a high amount of salts, sugars, proteins, lipids, and sometimes even water, can cause problems with the operation of analytical equipment. As a result, metabolites that are present in low concentrations may not be detected in the biological samples. Therefore, problems caused by the sample matrix need to be identified and resolved to ensure the acquisition of informative and accurate extracellular metabolite profiles [50]. There are several options for overcoming the difficulties instigated by the matrix of a biological sample, e.g.,

(a) liquid-liquid separation where metabolites of interest are separated into an immiscible solvent,
(b) using a column or solid-phase matrix to trap the metabolites, and
(c) selective solubilization, which is the complete evaporation of the solvent to concentrate the sample and the metabolites are then dissolved with suitable solvents.

**Figure 3:**
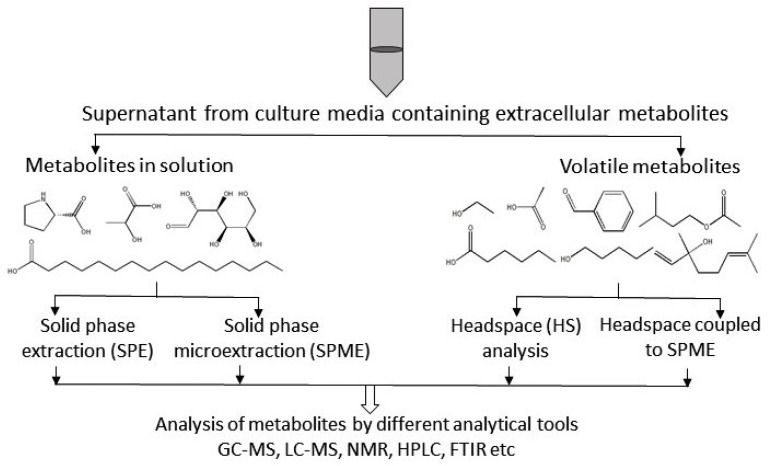
Overview of extracellular metabolite analysis. Different technical methods, such as solid-phase extraction (SPE), solid-phase micro extraction (SPME), head space (HS) analysis, and HS-SPME, are used for the preparation of extracellular samples. Sample preparation protocols depend on the type of metabolite. Here, GC-MS = gas-chromatography coupled to mass spectrometry; LC-MS = liquid-chromatography coupled to mass spectrometry; NMR = nuclear magnetic resonance spectroscopy; HPLC = high pressure liquid chromatography; FTIR = Fourier transform infra-red spectroscopy.

The use of solvents in the liquid-liquid separation of metabolites from extracellular media is very laborious and time consuming, making it unpopular for metabolite profiling. This approach is also known to result in incomplete phase separations and lower recovery rates. In addition, this type of separation also requires the use of large amounts of glassware and organic solvents. A better separation of metabolites, however, is achieved via solid-phase extraction to trap metabolites of interest [6] and selective solubilization is also a widely used technique for the analysis of extracellular metabolites in solution. However, there are two commonly used methods for the separation of targeted metabolites that are present in solution, namely solid-phase extraction (SPE) and solid-phase microextraction (SPME) (Figure 3). The technical details of these two separation procedures will be discussed in the next subsections.

#### 2.1.1. Solid-Phase Extraction

Solid-phase extraction (SPE) is a sample separation and extraction method, which causes the exhaustive removal of metabolites from a liquid sample resulting in targeted analytes being retained on a solid sorbent [51]. SPE is usually used to prepare liquid samples and for the extraction of semi-volatile and non-volatile metabolites. It is more efficient than liquid-liquid separation and is easier to perform. Both solid phase and liquid phase are used to isolate one type of analyte from a solution. SPE is widely used to concentrate the metabolites of interest, mainly for targeted analysis, when they are in very low concentrations [52]. Moreover, it is very useful to remove impurities and interfering matrix components from samples or to prepare the sample in solution for subsequent analysis [6].

Different types of SPE sorbent materials, such as silica, alkylated silica (e.g., C-18), carbon-based sorbents, ion exchange materials, polymer materials, and restricted access materials (RAM) are commercially available for different types of metabolite extraction. Mixed-mode sorbents are a newer development and are based on multiple retention mechanisms due to incorporating diverse ligands in the same sorbent [53].

The SPE cartridge, or stationary phase, is contained in a glass or plastic column above a frit or glass wool. The column has a frit on the top and there is a stopcock that controls the flow of solvent in the column. The cartridges are placed on a vacuum manifold which is used to control the solvent flow rate through the cartridge. Collection tubes are placed under the cartridge to collect the waste liquid after washing the column. During elution, clean tubes replace those waste tubes to collect the final sample containing the metabolites of interest [6]. The trapped metabolites in the SPE cartridge can then be eluted by using an appropriate extraction solvent of sufficient strength [53], selected on the basis of its polarity, pH, and mobile phase ionic strength [6].

Three different SPE stationary phases, such as normal, reversed, and ion-exchange, are available with SPE cartridges and disks. These stationary phases are mainly silica-based and commonly used in the preparation of samples for metabolomics analysis, allowing the separation of metabolites according to their chemical properties (Table 1).

● Normal Phase

A non-polar matrix and polar stationary phase are used to extract non-polar analytes. The retention of analytes depends on chemical interactions (e.g., hydrogen bonding, pi-pi interactions, dipole-dipole interactions, etc.) between functional groups of analytes and sorbents. The trapped analytes are eluted by a more polar solvent which is able to break up the binding mechanism.

● Reversed Phase

Reversed phase SPE has a mobile phase with a polar or moderately polar matrix and a non-polar stationary phase. This non-polar stationary phase is derivatized with hydrocarbon chains that allow the retention of non-polar metabolites because of the hydrophobic effect. Non-polar forces involved in this phase are known as van der Wals or dispersion forces. A non-polar solvent is also used to elute the analytes.

● Ion-Exchange Phase

Metabolites which are charged while in solution can be extracted by using an ion-exchange phase. The retention of analytes is due to the electrostatic attraction of the charged functional group of the metabolite to the charged group of the matrix. A solvent with a pH level that neutralizes these charged groups is used to elute the analytes.

SPE is a sensitive method that enables the simultaneous analysis of a number of metabolites by limiting possible ion suppression effects [54]. The main limitation of SPE in metabolomics is its selectivity. Thus, it is suitable for targeted analyses, but not for global metabolite profiling. While the use of SPE for extracellular metabolite extraction is not very common, it has been successfully applied to purify and identify antifungal compounds (phenyllacticacid, 4-hydroxyphenyllactic acid, benzoic acid, methylhydantoin, mevalonolactone, etc.) and some other small peptides from lactic acid bacteria [55,56]. This sample preparation technique has great potential for the extraction and purification of antibacterial compounds (small analytes) secreted into the extracellular media.

#### 2.1.2. Solid-Phase Microextraction

Solid-phase microextraction (SPME) is a portable small sample preparation technique invented by Pawliszyn and colleagues which is both fast and simple [57,58,59]. The development of an automated SPME method made this technique suitable for the high-throughput analysis of samples [60,61]. In SPME, a small amount of sample (<100 µL) is exposed for a specific time period to a small amount of extracting phase dispersed on a solid support allowing metabolite extraction. Two approaches are available. In one, a partitioning equilibrium occurs and it causes convection between the sample matrix and the extraction phase. The number of metabolites extracted is independent of convection conditions. The other approach uses a short-time pre-equilibrium where convection is constant and, as a result, the amount of extracted metabolite is time dependent [62].

The first SPME device consisted of coated fibers in a micro-syringe referred to as a fiber SPME. A polymer-coated fused-silica fiber was exposed to a sample for a predetermined time until equilibrium was reached. The fiber was then removed from the solution to desorb the analytes into the injector of an analytical instrument [63]. Different types of SPME coating are commercially available today, which are able to extract a wide range of metabolites [64].

Even though the use of SPME allows the separation of many metabolites, this technique has both advantages and disadvantages. For instance, it is resource efficient in that it does not require solvent for the elution of analytes and the fibers are reusable. Furthermore, SPME is portable so that it can be used in the field, as well as in the laboratory. However, the coated fibers are expensive, fragile, and have a limited extraction capacity, resulting in poor sensitivity [6,59]. Although SPME is a popular technique because of its simplicity, sensitivity, and reproducibility, the optimization of sampling conditions and set up of suitable experiments are required to obtain good quality data [65].

### 2.2. Metabolites in Gas Phase

Volatile compounds, or gaseous metabolites, present in extracellular samples provide precious information on different properties of microorganisms and/or their phenotypes and their metabolic pathways. Metabolites already in gas phase are easy to analyze by GC-MS and require no further derivatization (Figure 2). Thus, their analysis takes less time than non-volatile compounds. However, they are very difficult to collect due to their high diffusion rates and also because of their very low concentrations (sometimes near or below the detection limit) [6]. Thus, to improve their detection by analytical instruments, the metabolites must be captured and concentrated before analysis.

Several methods have been developed in recent years to trap and concentrate volatile compounds in extracellular samples. SPME methods using adsorptive materials, such as porous carbon, or sorptive polymers, are often used to analyze volatile compounds. Headspace analysis coupled with SPME is another alternative technique employed to analyze these compounds.

#### SPME and Headspace Analysis of Volatile Compounds

SPME is considered a promising sample preparation technique for the extraction of volatile compounds from microorganisms because of its ability to separate metabolites with a wide range of properties (both in liquid and solid samples) at a very low concentration (ppt to ppm) [66]. SPME and headspace analysis are often used in combination with GC-MS to analyze the metabolites in gas phase. Volatile compounds from microbial liquid cultures and fermentation flasks can easily be captured from the amount of headspace gas above the culture and they can then be detected and identified by GC-MS. This can be done in two ways:

1. Direct headspace analysis where volatile compounds are collected into a syringe and analyzed by GC.
2. Headspace SPME (HS-SPME) which is a coupled technique in which the gas sample in the headspace is trapped on the SPME fiber [6].

In headspace analysis, a liquid sample is collected and heated to vaporize in the headspace. Both manual and automated systems can be applied; however, automated systems show better reproducibility [67]. The headspace analysis of a microbial culture provides qualitative results about volatile compounds [68]. The microbial contamination of food can also be determined by collecting the volatile metabolites in the headspace gas and then analyzing those using GC-MS or an electronic nose [69]. HS-SPME is a popular solvent-free method employed for analyzing microbial samples. Volatile compounds are isolated and concentrated without any derivatization [70]. This fast, simple, and sensitive method yields highly reproducible results. Moreover, HS-SPME is more advantageous compared to direct SPME. For instance, it requires less time to extract the targeted metabolites due to the faster diffusion rate of the analytes in the gas phase. Moreover, matrix effects can also be reduced by using HS-SPME. Overall, the use of SPME along with HS provides highly reproducible results, which is obviously very useful for the analysis of metabolites in the gas phase.

## 3. Concentration of Extracellular Samples to Improve Detection

Sample concentration is an important aspect of metabolome analysis because metabolites are diluted in both intracellular and extracellular samples. Concentration of the samples is necessary before analysis by analytical tools to improve the limit of detections [47,71]. If the extracellular samples are concentrated, they can be derivatized or analyzed directly [47]. Lyophilization, or freeze drying, is commonly used to concentrate extracellular samples by the removal of water. Conversely, organic solvent evaporating systems or vacuum drying methods are also used to remove water from samples, although the thermal degradation of metabolites needs to be considered when these techniques are used. Non-aqueous extracellular samples are, however, concentrated using solvent evaporation techniques.

### 3.1. Freeze-Drying

Freeze-drying is a widely used sample concentration method in metabolomics where samples are immediately frozen and then dried by sublimation to remove all of the frozen solvents. Freeze-drying is considered a non-destructive sample concentration and dehydration process that avoids the thermal degradation of compounds. A variety of manual and completely automated freeze-dryers are commercially available these days, including facilities to dry samples under a very low temperature (–65 °C). Some “smart” freeze-drying instruments, for example, a manometric temperature measurement (MTM), measure the metabolite temperature by rapidly isolating the freeze-drying chamber from the condenser while also monitoring the pressure [72]. Recent developments in freeze-drying techniques are very useful and efficient for concentrating samples for both intracellular and extracellular metabolite analysis [73]. It is, however, a slow and complex procedure that may be not be suitable for metabolites that are susceptible to oxidation. To overcome the oxidative degradation of metabolites, inert gas (e.g., nitrogen) must be used to break the vacuum [47].

The nature of the metabolites and the sample matrix need to be considered carefully while concentrating the metabolomics samples. Moreover, appropriate precautions also need to be followed to overcome problems, e.g., the thermal and oxidative degradation of labile metabolites. Factors such as the solution pH and sample matrix are very important and these may cause problems while freeze-drying the samples. For instance, extracellular samples with a high sugar concentration are almost impossible to dry completely, leaving only sticky undried samples. To avoid this, such samples must be diluted prior to freeze-drying. Moreover, samples containing a high volume of organic solvent cannot be frozen and must have water added to increase the water:solvent ratio. This then enables the sample to be frozen but also increases the drying time. After freeze-drying, another issue is the resuspension of samples because metabolite losses may occur due to the incomplete recovery of the dried samples [74,75]. However, besides having some minor disadvantages, freeze-drying is still a method of choice for many laboratories because of its efficiency in terms of the sample concentration.

### 3.2. Vacuum-Drying

The vaccum-drying procedure using a speed-vac instrument makes use of a combination of the centrifugal force, heat, and vacuum to remove moisture from a given sample. This process dries a sample through evaporation, mainly by converting the liquid to vapor. It is mostly used to concentrate a small amount of sample to complete dryness. Therefore, vacuum-drying, being faster and comparatively less aggressive than freeze-drying, is preferable in some cases [6]. For instance, the reagents for trimethyl silyl derivatization are extremely sensitive to moisture, and thus, the vacuum-drying of samples is often preferable [26,36]. However, vacuum-drying often requires the use of heat; therefore, thermos-labile metabolites might be degraded during the process. Sometimes, the addition of extra solvent (e.g., methanol) is also required, which assists in the fast evaporation of samples [76].

## 4. Storage of Extracellular Microbial Samples

Once extracellular samples have been extracted and concentrated, the next step is to store them under appropriate conditions prior to analysis. To avoid any unwanted changes during the storage, necessary precautions need to be undertaken. Storage conditions and time are, however, dependent on the types and stability of metabolites. Even though metabolomic samples are quenched before the separation and extraction of extracellular metabolites, it is better to ensure that no chemical reaction is taking place during the storage. Therefore, it is highly recommended that the extracellular samples must be stored in the dark and at a very low temperatures (e.g., −20 °C, −80 °C) to avoid the degradation caused by light and heat. This way, it is possible to retain the original properties of metabolites.

Some metabolites can lose their functionality during storage at low temperature and can be oxidized easily, and this may completely alter their characteristics. To protect them, they must be kept under vacuum [6]. Stable metabolites (i.e., do not oxidize and are unaffected by exposure to normal temperature) can be stored at room temperature and mostly, the samples concentrated to powder that do not contain any water are suitable for storage under vacuum at room temperature. However, this is not a common practice in metabolomic laboratories. Setting up appropriate storage conditions for all types of metabolomic samples is a primary responsibility of the laboratories in order to generate reproducible and high-quality data.

## 5. Integration of Extracellular Metabolomics Data to Genome Scale Metabolic Models

The genome-scale reconstruction of entire organisms and modelling of their physiology in a predictive manner is a comparatively new approach and has been successfully used in systems metabolic engineering to design microbial cell factories [77,78,79,80,81,82]. This is an emerging interdisciplinary field that makes use of a systems-level view of an organism to determine the genotype-phenotype relationship, without the need for kinetic parameters [83,84]. The network is explored by imposing physico-chemical constraints (e.g., omics data, cell growth) and by testing the cell efficiency under optimality criteria [85]. The high amount of data generated from different omics approaches, especially from metabolomic studies, can now be integrated into a genome-scale model to gain novel insights on microbial metabolism [86].

Recent developments in profiling extracellular metabolites are now allowing us to gather a large amount of information on microbial systems. These extracellular metabolites are either present in the culture media or secreted by the microorganisms during growth due to experimental perturbations. Therefore, these data contain valuable information about the cell metabolism and how the environment can affect the utilization and production of various metabolites, indicating the alteration of intracellular status. The generation of intracellular metabolites data is comparatively troublesome, mainly because of the need to rupture the cell in order to release the metabolites [9,26,86]. Therefore, the integration of extracellular metabolomics data into metabolic models is clearly an advantageous approach.

The integration of extracellular metabolomics data into genome-scale models is now allowing the prediction of the intracellular status of microorganisms. For instance, Cakir et al. (2007) incorporated small-scale extracellular metabolomics data with a genome-scale metabolic model of yeast to predict the oxygen consumption and ethanol production ability of mutant strains with respiratory deficiencies [87]. A few years later, Mo et al. (2009) published a study that integrated a large extracellular metabolite profiling data into a highly curated genome-scale metabolic model of *Saccharomyces cerevisiae* iMM904 to determine how the variation in extracellular metabolite levels is associated with changes in intracellular metabolites fluxes [86]. They also successfully validated the predictions generated from the model.

A comprehensive and detailed protocol entitled “MetaboTools” is now available for integrating extracellular metabolomics data obtained from microorganisms, plants, and animals [39]. This toolbox integrates the extracellular metabolite profiles into the network context and generates contextualized models that contain a subset of metabolic models. These metabolic models are designed to predict the intracellular pathways based on the extracellular metabolites by determining the utilization and production of different metabolites under different environmental conditions [38,39]. Although this approach of integrating extracellular metabolomics data into metabolic models and the prediction of intracellular status is gaining popularity, a comprehensive status of microbial metabolism can only be obtained by combining both intra- and extracellular metabolomic datasets [37].

## 6. Conclusions

The analysis of extracellular metabolites from a microbial culture provides crucial information about the intracellular status of a microbial system that occurs in response to the surrounding environment. Therefore, this approach has already been used widely in both fundamental and applied research [88,89,90,91]. However, there is still scope to improve the identification and quantification of metabolites in a wide range of biological samples and it would be useful for the analysis of both extracellular and intracellular samples. The sets of information gained from both intracellular and extracellular microbial samples are complementary to each other, which will allow an understanding of the complete overview of microbial metabolism.

## Figures and Tables

**Table 1:** Comparison among different solid-phase extraction phases.

	Phase		
Parameters	Normal	Reversed	Ion-Exchange
Solvent polarity	High	Low	High
Range of solvent polarity	Low to medium	High to medium	High
Solvents for elution	Acetone, ethyl acetate	Water/methanol/acetonitrile solution	Salts and buffers
Loading solvents	Toluene, hexane	Water and buffers	Water and buffers
Eluted sample	Less polar	Most polar	Weakly ionized
